# Cow-to-mouse fecal transplantations suggest intestinal microbiome as one cause of mastitis

**DOI:** 10.1186/s40168-018-0578-1

**Published:** 2018-11-08

**Authors:** Chen Ma, Zheng Sun, Benhua Zeng, Shi Huang, Jie Zhao, Yong Zhang, Xiaoquan Su, Jian Xu, Hong Wei, Heping Zhang

**Affiliations:** 1 Key Laboratory of Dairy Biotechnology and Engineering, Inner Mongolia Agricultural University, Hohhot, 010018 China; 2 Department of Laboratory Animal Science, College of Basic Medical Sciences, Third Military Medical University, Chongqing, 400038 China; 3 Single-Cell Center, CAS Key Laboratory of Biofuels and Shandong Key Laboratory of Energy Genetics, Qingdao Institute of BioEnergy and Bioprocess Technology, Chinese Academy of Sciences, Qingdao, 266101 Shandong China; 4 The Engineering Technology Research Center for Germ-free and Genome-editing Animal, Huazhong Agricultural University, Wuhan, 430070 People’s Republic of China

**Keywords:** Mastitis, Intestinal microbiota, Fecal microbiota transplantation, Germ-free mice, Probiotics

## Abstract

**Background:**

Mastitis, which affects nearly all lactating mammals including human, is generally thought to be caused by local infection of the mammary glands. For treatment, antibiotics are commonly prescribed, which however are of concern in both treatment efficacy and neonate safety. Here, using bovine mastitis which is the most costly disease in the dairy industry as a model, we showed that intestinal microbiota alone can lead to mastitis.

**Results:**

Fecal microbiota transplantation (FMT) from mastitis, but not healthy cows, to germ-free (GF) mice resulted in mastitis symptoms in mammary gland and inflammations in serum, spleen, and colon. Probiotic intake in parallel with FMT from diseased cows led to relieved mastitis symptoms in mice, by shifting the murine intestinal microbiota to a state that is functionally distinct from either healthy or diseased microbiota yet structurally similar to the latter. Despite conservation in mastitis symptoms, diseased cows and mice shared few mastitis-associated bacterial organismal or functional markers, suggesting striking divergence in mastitis-associated intestinal microbiota among lactating mammals. Moreover, an “amplification effect” of disease-health distinction in both microbiota structure and function was apparent during the cow-to-mouse FMT.

**Conclusions:**

Hence, dysbiosis of intestinal microbiota may be one cause of mastitis, and probiotics that restore intestinal microbiota function are an effective and safe strategy to treat mastitis.

**Electronic supplementary material:**

The online version of this article (10.1186/s40168-018-0578-1) contains supplementary material, which is available to authorized users.

## Background

Mastitis is a potentially fatal inflammatory reaction of the parenchyma of the mammary gland and affects nearly all lactating mammals [1]. In human, mastitis carries significant health and social burden: globally, ~ 10% of breastfeeding women suffered from the local pain, redness, swelling, and warmth of mammary gland and systemic symptoms like fever and abscess, which can traumatize both the mothers and their neonates [2]. In cattle, mastitis is the most common and costly disease in the dairy industry [3]. Swelling and pain in the udder and systemic involvement such as fever, anorexia, and shock detriment compromise animal well-being [4] and halt their productivity due to the high levels of leukocytes or residual antibiotics in milk.

Despite decades of research into the cause of hominid and bovine mastitis, whether there are specific pathogens behind the disease remains not entirely clear, with evidence from various studies pointing to different direction [5, 6]. Mastitis can occur when opportunistic pathogens, frequently members of the normal host microbiota, invade and colonize the mammary gland [7, 8]. On the other hand, lines of evidence have argued against the notion of specific pathogens as cause of mastitis. For example, although common members of the natural udder or breast microbiota such as *Staphylococcus aureus*, *Streptococcus* spp., and *Escherichia coli* are frequently present and associated with mastitis in udder and breast tissues, none of them are found in all bovine mastitis cases and none of them are found infectious from cows to cows [9, 10]. In particular, although its introduction via intramammary inoculation into cows resulted in clinical mastitis (exposure to ultra high dose of *Streptococcus uberis*), *Streptococcus uberis* is also present in udder tissues of healthy cows [11, 12]. Thus, it is possible that factors other than these breast-associated bacteria, such as dysbiosis of gut microbiota, also play a role in mastitis etiology.

Here, using bovine mastitis as a model, we showed that dysbiosis of intestinal microbiota can lead to mastitis. Fecal microbiota transplantation (FMT) from mastitis cows, but not from healthy cows, to germ-free (GF) mice resulted in mastitis symptoms in mammary gland as well as inflammations in a wide range of tissues including serum, spleen, and colon in the mice. Probiotic intake in parallel with inoculation of fecal microbiota from diseased cows led to greatly relieved mastitis symptoms in the recipient mice, while intervention of probiotic shifted the murine intestinal microbiota to a state that is functionally distinct from both diseased and healthy microbiota yet structurally similar to diseased microbiota. Despite the conservation in mastitis symptoms, diseased cows and mice shared few mastitis-associated bacterial organismal or functional markers, suggesting a high degree of divergence in mastitis-causing intestinal microbiota among lactating mammals. On the other hand, an “amplification effect” of disease-health difference in both microbiota structure and function is apparent during the FMT from cow to mouse. Therefore, dysbiosis of intestinal microbiota is one cause of mastitis, and probiotics that target restoration of intestinal microbiota function are an effective and patient-friendly strategy to treat mastitis.

## Results

### Intestinal microbiota of mastitis cows are distinct from those of healthy cows

**Fig. 1:**
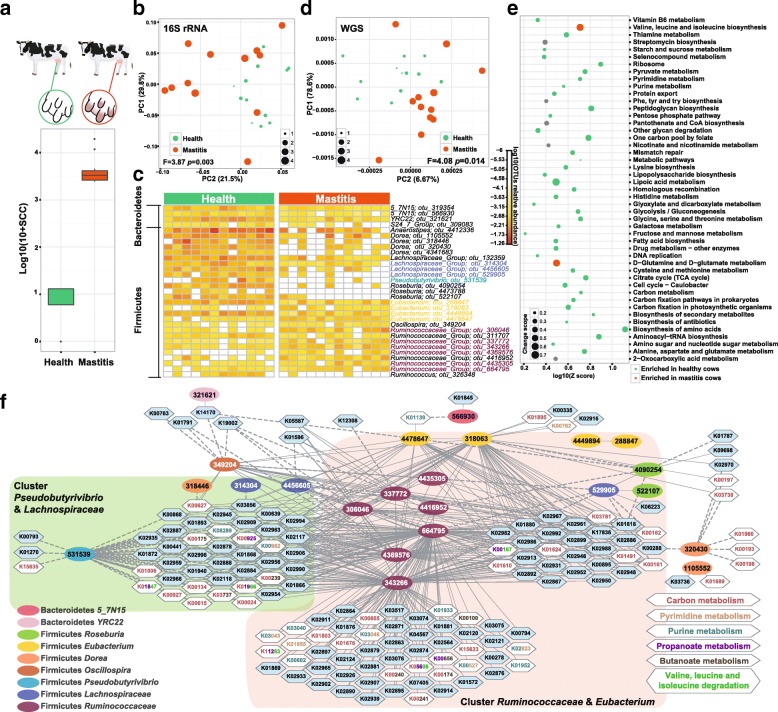
Distinction between healthy and mastitis intestinal microbiota in cow. **a** Comparison of somatic cell count (SCC) between healthy and mastitis cows (logarithmic base of 10). **b** Principal coordinate analysis (PCoA) via Meta-Storm distances: distinct organismal composition between healthy (green) and mastitis (red) cows was revealed. Size of dots represents somatic cell count (logarithmic base of 10). *F* value is the PERMANOVA result of β-diversity. **c** Heatmap showing the 31 discriminating operational taxonomic units (OTUs) between healthy and mastitis cows (abundance shown as logarithm base of 10). **d** Principal component analysis (PCA) of functional genes (annotated by KEGG) between healthy and mastitis cows. **e** KEGG metabolic pathways that differentiate the healthy and diseased state are shown as a line map (red dots: enriched in mastitis cows; green dots: enriched in healthy controls; size of the dots: value of identified KO number divided by all KOs number in the specific pathway). **f** Correlation network of differential OTUs and KOs, with elliptical nodes (OTUs) colored based on genus, edges defined by the type of correlation (dash: negative; solid: positive) and hexagonal nodes (KOs) colored (blue or white) based on pathways

To probe the link between gut microbiota and bovine mastitis, fecal microbiota from twelve 3–6-year-old cows diagnosed of mastitis were compared to twelve physically similar, age-matched, healthy cows that served as the control (Additional file 1: Table S1; Materials and method). Disease status was classified based on milk somatic cell count (SCC) plus clinical signs that include abnormal milk production and udder redness and swelling (Materials and method): average SCC of the diseased cows was 715-fold that of the healthy ones (Fig. 1a).

Between the healthy and mastitis cows, full-length 16S rRNA sequencing of stool DNA by the PacBio platform revealed identical α-diversity (via Shannon Index [13]), yet significant difference in β-diversity (via Meta-storms distance [14, 15]; *F* = 3.87, *p* = 0.003, PERMANOVA; Fig. 1b). Thirty-one bacterial operational taxonomic units (OTUs) (10 positively and 21 negatively correlated; adjusted *p* < 0.01, Wilcoxon rand-sum test; Additional file 2: Table S2A, Fig. 1c) were significantly associated with bovine mastitis: all are from the phyla of Firmicutes (*Anaerostipes*, *Dorea*, *Lachnospiraceae*, and *Roseburia* enriched in healthy cows, while *Oscillospira* and *Ruminococcaceae* enriched in mastitis) and Bacteroidetes (all enriched in healthy cows).

To identify mastitis-associated functional genes, 24 stool samples from the 12 sick and 12 healthy cows were each shotgun sequenced and compared based on profiles of encoded bacterial genes (Materials and method). Significant difference in β-diversity was also observed between the diseased and healthy cows (*F* = 4.08, *p* = 0.014, PERMANOVA, Fig. 1d), suggesting that in bovine mastitis both organismal and functional structure were altered in the gut microbiota. Biomarker analysis revealed 269 positively (*n* = 86) or negatively (*n* = 183) mastitis-associated KEGG Orthology (KO) (*p* < 0.05; Wilcoxon rank-sum test, Additional file 3: Table S3A, C), which were enriched in 42 metabolic pathways (*Z* > 1.6; Materials and method; Additional file 3: Table S3F). In mastitis cows, two pathways of (i) valine, leucine, and isoleucine biosynthesis and (ii) d-glutamine and d-glutamate metabolism were enriched, while the majority, i.e., the remaining 40 pathways, were depleted (Fig. 1e). The latter included vitamin B-related metabolic pathways, i.e., lipoic acid metabolism (all the three identified KOs in this pathway were less abundant, abbreviated as 3/3), vitamin B_6_ (5/7), one carbon pool by folate (15/16), and thiamine metabolism (8/10). Vitamin B as a cofactor for many biochemical reactions may suppress inflammation [16], yet intestinal microbes are a major source of vitamin B in human and other mammalian hosts who are unable to synthesize them [17]. Thus, it is possible that mastitis is associated with a disorder of vitamin B metabolism in intestinal microbiota, which may deserve further investigation.

Pathways with lower abundance in mastitis cows, which presumably limit inflammation and protect intestinal mucosa, also included, e.g., lysine biosynthesis (13/16), fatty-acid biosynthesis (9/11), purine (45/60), and pyrimidine (41/49) metabolism (increased levels of the purine metabolite inosine can inhibit multi-organ inflammation in mice [18]) and selenocompound metabolism (6/9). Carbon metabolism including pyruvate metabolism (23/34), galactose metabolism (14/21), citrate cycle (18/24), and glycolysis/gluconeogenesis (22/32) was also less abundant, suggesting reduced carbon metabolic activity of gut microbiota in mastitis cows.

To probe the link between the mastitis-associated organisms and functional genes, a taxon-function interaction network was constructed based on abundance pattern of the disease-associated OTUs and KOs in the 24 animals [19] (Fig. 1f), where the edges that connect OTU nodes to all those KOs with whom linear correlation was found (Spearman correlation coefficient > 0.8, adjusted *p* < 0.01) indicate potential organism-function links. Two prominent OTU-clusters, all from Firmicutes Phylum and together accounting for 42% of disease-associated OTU, were found: one around *Ruminococcaceae* and *Eubacterium* and the other around *Pseudobutyrivibrio* and *Lachnospiraceae*. These OTUs are all positively or negatively correlated with carbon metabolism (Fig. 1f; Additional file 4: Table S4A). Specifically, *Ruminococcaceae* and *Eubacterium*, both enriched in mastitis, exhibit positive correlation with 121 KOs in propanoate (7/16; the number of KOs assigned to this pathways in the cluster/all identified KOs in this pathway) and butanoate (5/15) metabolism; purine (9/60) and pyrimidine (7/49) metabolism; and valine, leucine, and isoleucine degradation (5/12), suggesting implication of the activities from these Firmicutes in mastitis. Interestingly, a large functional-gene cluster of 41 KOs was positively correlated with *Eubacterium* and *Ruminococcaceae* while also negatively correlated with *Pseudobutyrivibrio* and *Lachnospiraceae* (both of which depleted in mastitis). This suggests that the shift in fine balance between these two specific OTU-clusters might underlie the health-to-mastitis conversion, where enrichment of *Eubacterium* and *Ruminococcaceae* and depletion of *Pseudobutyrivibrio* and *Lachnospiraceae* may result in upregulation of propanoate and butanoate metabolism, purine metabolism, valine, leucine, and isoleucine degradation.

### Intestinal microbiota from mastitis cows, but not healthy cows, induced mastitis in germ-free mice, whereas probiotic intake alleviated mastitis

To test whether the structural and functional alteration of gut microbiota are a cause or a consequence of bovine mastitis, fecal microbiota from the 12 mastitis and 12 healthy cows were respectively pooled and then inoculated into adult, pregnant gnotobiotic mice via fecal microbiome transplantation (FMT; Materials and method). Among the 35 recipient mice, 11 underwent FMT from healthy cows (group H), 12 from mastitis cows (group M), while a third group of 12 (group P) was established where the mice underwent both FMT from mastitis cows and a 25-day regimen of probiotics intake after FMT (via intragastric administration of 5 × 10^8^ cfu/day *Lactobacillus casei*; Materials and method).

**Fig. 2:**
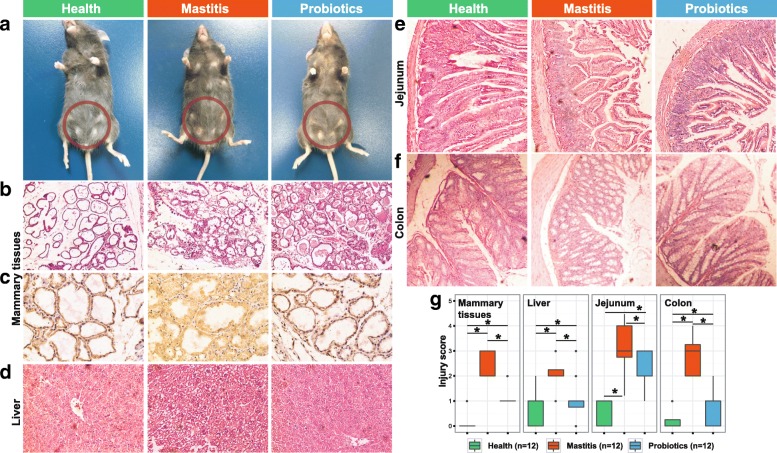
Histological analysis of mouse tissues after FMT and probiotics intervention. **a** Pathological changes in mammary gland surface, where two abdominal mammary glands were swelling in the mastitis group of mice on day 25 after FMT. Breast of mice was highlighted by red circles. **b** Representative photomicrographs of hematoxylin-eosin stained mammary gland tissue (× 200 magnification). **c** CD45 immunohistochemical staining sections at × 400 magnification. **d**–**f** Representative photomicrographs of hematoxylin-eosin stained liver (× 200), jejunum (× 100), and colon tissue (× 100). **g** The injury score of mammary gland, liver, jejunum, and colon

Comparison of murine post-FMT inflammatory responses among the three groups revealed that gut microbiota from mastitis cows induced a much greater inflammatory (of mammary gland, liver, jejunum, and colon) response than those from healthy cows. On mammary gland surface, severe inflammation that corresponded to mastitis was observed in group M, yet no pathological changes were visually apparent in groups H or P (Fig. 2a). This was supported by histopathologic section that evaluates mammary gland tissue damage (e.g., mammary alveolus thickening, hyperemia, and edema) and extent of inflammatory cell infiltration (i.e., stained leukocyte cells) [20, 21]. For example, under hematoxylin-eosin (HE) staining, group M featured broken lobules of the mammary gland, damaged acinuses, and destroyed epithelial cells, with inflammatory cells including macrophages, neutrophils, and blood cells detected in the mammary lobule (Fig. 2b); in contrast, in group H, no pathological changes were apparent, while in group P the lobules were largely complete and the acinuses were mostly intact (suggesting mitigated histopathology). These findings were further supported by the increased immunohistochemical staining of mammary gland for CD45 in group M (CD45 as the first and prototypic receptor-like protein tyrosine phosphatase is expressed on all nucleated hematopoietic cells and plays a central role in adaptive immunity [22]): inflammatory cells such as macrophages and neutrophils were found in group M but not in groups H or P (i.e., suggesting an inflammatory response in group M; Fig. 2c). The observed migration of leukocytes from blood into mammary gland indicated a bacteria-induced cellular inflammatory response that was stimulated by secreted chemotactic and inflammatory mediators [23].

The murine inflammation induced by diseased bovine intestinal microbiota seemed pervasive. HE staining of murine liver sections revealed blur of hepatic lobe, hyperemia, and ballooning degeneration of hepatocyte in group M, in contrast to the normal liver structures in group H and the recovered liver structures in group P (Fig. 2d). Pathological section of murine intestinal and colon revealed in group M severe disorder in mucosa structure (necrosis of epithelial cells, extension of the subepithelial space, and structural damage of villi); in contrast, group H mice exhibited normal intestinal mucosa with integral villi, while probiotics intake in group P significantly improved intestinal and colon histology, featuring alleviated swelling of mucosa, less subepithelial space expansion, and well-arranged villi structure (Fig. 2e, f). In fact, for each of the tissues tested, pathological grade of injury was significantly higher in group M than in either group H (*p* < 0.01) or group P (*p* < 0.01; Fig. 2g). To test whether bacteria on the breast surface can induce mastitis, three mice were transplanted with healthy cow feces and were administered with mastitis cow feces on the surface of their breast (Materials and Method). HE staining showed that no inflammation was present in the mammary glands of the three mice throughput the duration of experiment (Additional file 5: Figure S1).

**Fig. 3:**
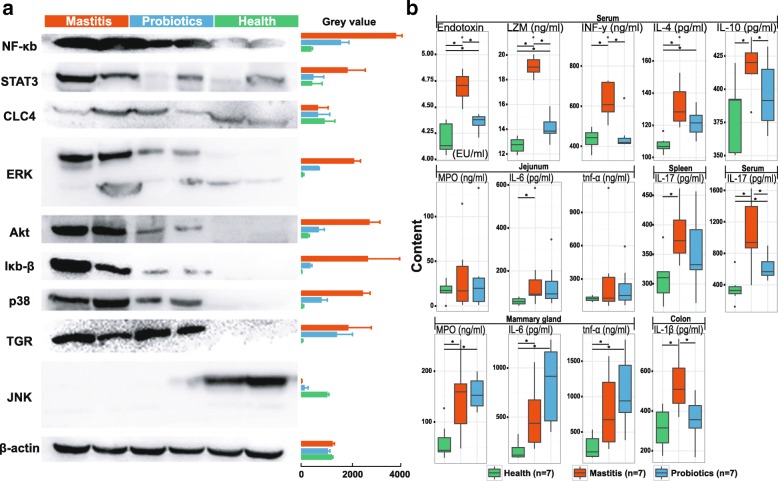
Effect of probiotics (*Lactobacillus casei*) administration on mice that were predisposed to risk of mastitis. **a** Western blots for quantification of NF-κb, Iκb-β, ERK, p38, JNK, STAT3, TGR, CLC4, and Akt protein levels in mammary glands (*n* = 2 per group), with β-actin as internal control. **b** Quantification of inflammatory cytokines in various tissues or organs using ELISA (*n* = 7 per group). The assays were all performed for the three groups of mice at Day 25 after FMT. Asterisk indicates significant difference between two groups (*p* < 0.05; Student’s *t* test)

To assess the activation of immunological signaling pathways in the murine mammary gland, a panel of nine key cytokines were assayed by Western blot at day 25th after FMT (Fig. 3a; the housekeeping gene of β-actin as control), which includes NF-κb and Iκb-β in the NF-κb signaling pathway; ERK, p38, and JNK in the MAPKs signaling pathway; DNA binding protein STAT3 (which responds to epidermal growth factor production and IL-6 secretion [24, 25]); membrane-bound bile acid receptor TGR (involved in regulating energy homeostasis and glucose metabolism [26]); CLC4 (essential regulator of cell volume and repair of epithelial damages [27]); and Akt (which phosphorylates and inhibits proapoptotic components of the intrinsic cell death machinery [28]). Group M reported much higher levels for seven of the nine cytokines than group H (e.g., NF-κB is 9.12-fold higher in Group M), except CLC4 (equivalent) and JNK (29.4-fold lower in Group M). In group P, levels of the cytokines fell between group M and group H (except CLC4 which showed little variation); notably, the 57.7%-lower NF-κB level in group P than group M indicated an anti-inflammatory effect of probiotics that is linked to inhibition of NF-κB pathway activation (Fig. 3a).

Furthermore, a number of murine inflammatory cytokines produced predominantly by activated macrophages [21], including tumor necrosis factor (TNF), interferon (INF), myeloperoxidase (MPO), and interleukins (IL), were assayed via ELISA in various murine tissues: (i) TNF-α, MPO, and IL-6 in mammary gland; (ii) IFN-γ, IL-4, IL-10, IL-17, lysozyme, and endotoxin in serum; (iii) IL-1β in colon; (iv) IL-6 and TNF-α in jejuna; and (v) IL-17 in spleen (Fig. 3b; Additional file 6: Table S5). Compared to group H, group M mice exhibited increased level of all the cytokines tested (*p* < 0.01), consistent with a much higher post-FMT inflammatory response in this group. Interestingly, for the majority of cytokines tested, their level in group P was higher than group H yet lower than group M, with the notable exceptions being at the mammary tissues, where MPO, IL-6, and TNF-α in group P were higher than or equivalent to those in group M (Fig. 3b). Considering that group P exhibited mitigated histopathology (plus absence of macrophages and neutrophils) in the murine mammary gland and reduced pathological grade of injury in other organs, upregulation of cytokine secretion (a key feature of augmented immune protection) that resulted from probiotics administration may have underlie the alleviated mastitis symptoms in group P. Moreover, the highest level of serum endotoxin found in group M suggested the possibility of access of gut bacteria to the blood system through hepatoenteral circulation, which contributed to the mammary gland inflammation.

### Organismal and functional distinction of intestinal microbiota between health and mastitis hosts was amplified by the cow-to-mouse FMT

**Fig. 4:**
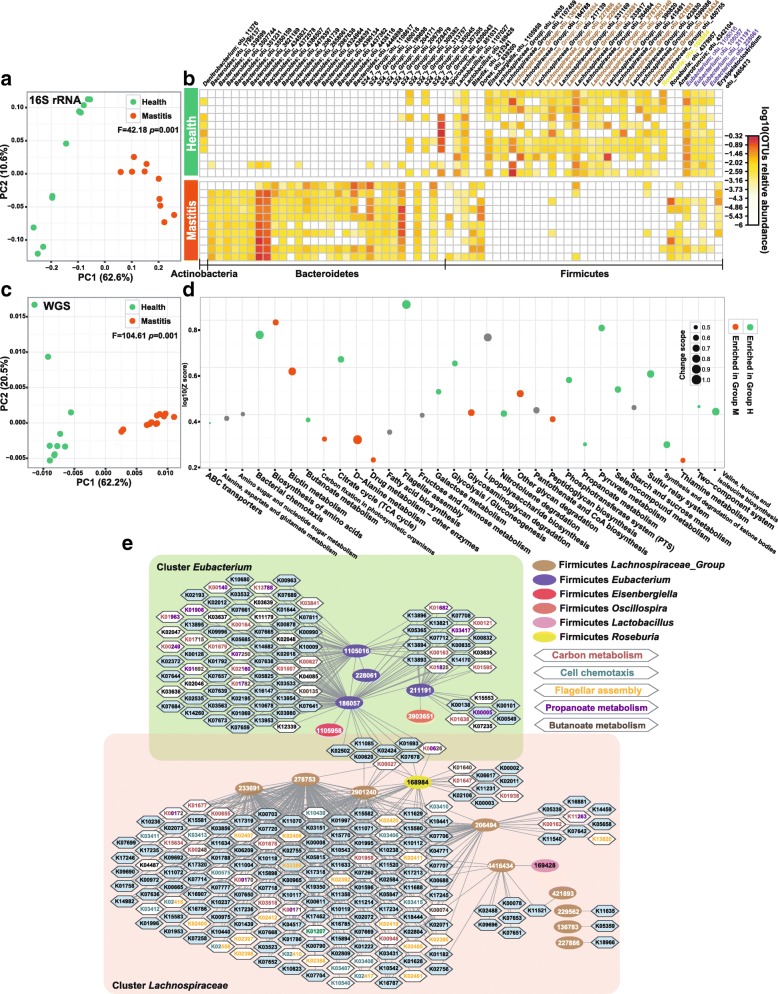
Distinction between healthy and mastitis intestinal microbiota in the mice after FMT. **a** PCoA clustering of the organismal structure of microbiota based on Meta-Storm distance. Percentage of variation explained by each principal coordinate is indicated on the axes. **b** Heat map of the 66 differential OTUs between group M and group H of mice. Relative abundance was shown as log 10 based. **c** PCA of functional gene structure between group M and group H of mice. **d** Significantly changed pathways of murine gut microbiota between group M and group H of mice. **e** Correlation network of differential OTUs and KOs, which revealed the taxon-function links

To mechanistically probe the distinct disease outcome among the three post-FMT murine groups, both full-length 16S rRNA gene amplicons and shotgun metagenomes were analyzed for stools of each of the 35 mice at day 25th after FMT (Materials and Method). The 16S rRNA amplicon analysis revealed that, despite identical α-diversity, distinction in β-diversity between group H and group M mice was highly significant and in fact much greater (*F* = 42.19, *p* = 0.001; PERMANOVA, Fig. 4a) than that between diseased and healthy cows (*F* = 3.87, Fig. 1b). Underlying the high degree of discrimination are 66 OTUs (Additional file 2: Table S2B) from the phyla of Firmicutes (35 of them), Bacteroidetes (30), and Actinobacteria (1). Most of the Firmicutes OTUs (e.g., those from *Lactobacillus*, *Eisenbergiella*, *Lachnospiraceae_Group*, and *Eubacterium* genera) were enriched in group H, yet most of the Bacteroidetes OTUs enriched in group M (Fig. 4b).

Comparison of shotgun metagenomes, i.e., β-diversity based on encoded microbial genes (via cosine distance of KOs), suggested strong functional discrimination of group M from group H mice (*F* = 104.61, *p* = 0.001, Fig. 4c). Moreover, the taxon- and function-based schemes are highly consistent (*p* = 0.0004, Monte Carlo test; Procrustes analysis based on PC1 and PC2). Among the 25 discriminating pathways (from 3525 differentiating KOs; Fig. 4d, Additional file 3: Table S3B, D, G), 9 were enriched while 16 depleted in group M (as compared to Group H). The most prominent is the lower abundance in mastitis of bacterial chemotaxis (23/24; depleted KOs vs all KOs in the pathway) and flagellar assembly (35/36), as well as intestinal mucosa repair and pathogen resistance, i.e., propanoate metabolism (32/44), butanoate metabolism (37/46), and selenocompound metabolism (13/21). Consistent with the findings in bovine, pyruvate metabolism (23/34), galactose metabolism (14/21), citrate cycle (18/24), and glycolysis/gluconeogenesis (22/32) were all depleted in mastitis mice. However, contrary to bovine, those enriched in mastitis mice included two vitamin B pathways of thiamine (9/14) and biotin (10/16) metabolism, as well as the degradation pathways of glycosaminoglycan (10/11) and other glycans (11/14). Considering the anti-inflammatory effect of glycosaminoglycan in rat arthritis [29], these results suggest that murine mastitis (but not bovine) may be potentially linked to the reduction of glycosaminoglycan, which is caused by the higher degradative activity of murine microbiota.

The murine OTU-KO correlation network, by correlating between the mastitis-associated taxonomical and functional profiles of the murine fecal microbiota, revealed two prominent clusters (Fig. 4e; Additional file 4: Table S4B): one around *Eubacterium* which is positively linked to propanoate metabolism (18/44; KOs from the clusters/all identified KOs) and butanoate metabolism (13/46), and the other around *Lachnospiraceae* (relative abundance of these OTUs all decreased in group M mice) which was positively associated with bacterial chemotaxis (15/24) and flagellar assembly (20/36). Notably, *Eubacterium* OTUs stood out in both of the murine and bovine OTU-KO networks, yet their taxonomical identity and associated KOs (i.e., functional roles) were both distinct (Fig. 1f): in cow, these OTUs were linked to the purine and pyrimidine metabolism, yet in mouse a different set of *Eubacterium* OTUs was linked to propanoate and butanoate metabolism. Thus the same bacterial components can exhibit distinct functions within cow and mouse.

### Mechanism of mastitis alleviation as induced by probiotics intake in mice

**Fig. 5:**
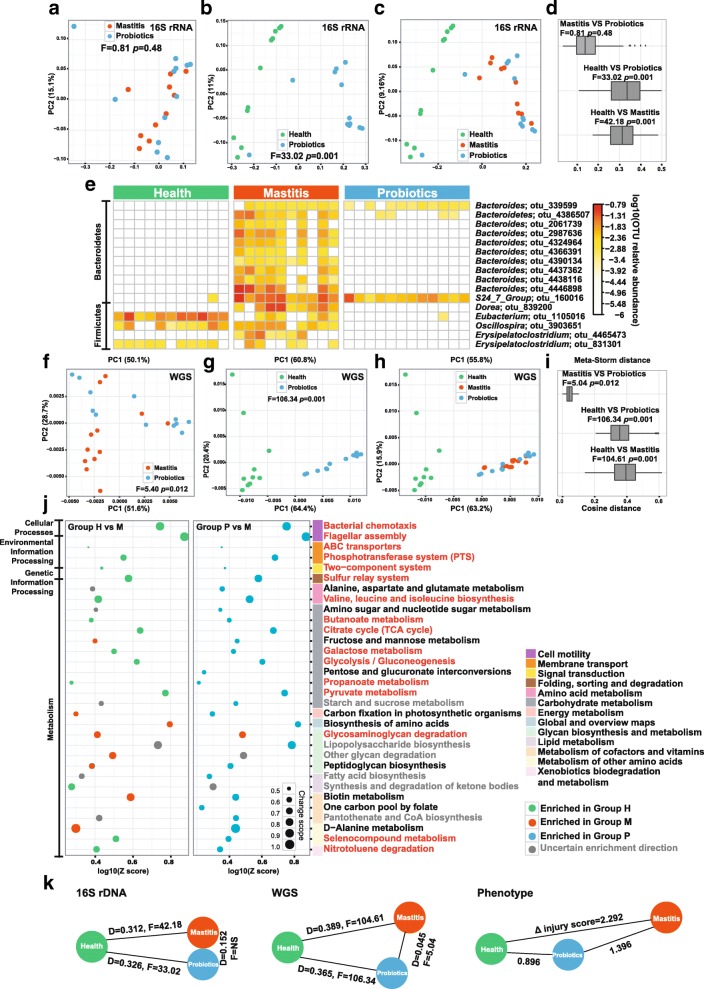
Influence of probiotics administration on structure and function of murine intestinal microbiota. **a**~**c** PCoA of organismal structures of microbiota among the three groups of mice. **d** Similarity of the microbiota in organismal structure based on Meta-Storm distance. **e**~**g** PCA of functional gene structure (based on KEGG annotation) among the three groups of mice. **h** Similarity of the microbiota in functional gene structure based on cosine distance of KOs. **i** Heat map of the 16 differential OTUs between group P and group M of mice. **j** Metabolic pathways that were significantly altered between group P and group M, and between group M and group H. Pathways that drove the microbiota toward healthy state after probiotics administration were highlighted via red font. Pathways upregulated in group P (as compared to group M) yet downregulated in group H (as compared to group M) were colored with black, which represent microbial pathways induced by probiotics intake yet did not drive the microbiota towards the healthy state. Those pathways that were altered in one comparison yet not in the other were colored as gray. **k** Degree of microbiota divergence among group P, group M, and group H of mice, in terms of organismal structure of microbiota, functional gene structure of microbiota, as well as the mastitis symptom of the host

Although probiotics intake in parallel with FMT from diseased cows resulted in a significant relief of mastitis, taxonomical structures of group P microbiota were indistinguishable from those of group M (*F* = 0.81, *p* = 0.48; PERMANOVA, Fig. 5a), yet are distinct from those of group H (*F* = 33.02, *p* = 0.001; Fig. 5b). Indeed, taxonomical structure of group P is much more similar to group M than to group H (Fig. 5c, d). In group P, 16 OTUs were significantly changed (all with lower abundance) as compared to group M: 11 from *Bacteroides* and *S24*_*7* (Bacteroidetes) and 5 from the genera of *Dorea*, *Eubacterium*, *Oscillospira*, and *Erysipelatoclostridium* (Fig. 5e; Additional file 2: Table S2C). Fifteen of these 16 OTUs (except OTU1105016) were also found in group H and depleted as compared to group M, suggesting in group P microbiota a certain degree of “recovery” in structure from the mastitis state to the healthy state.

Interestingly, the KO profile derived from shotgun metagenomes separated group P from either group M (*F* = 5.40, *p* = 0.012; Fig. 5f) or group H (*F* = 106.34, *p* = 0.001; Fig. 5g); however, consistent with the taxonomy-based relationship, group P is also more similar to group M than to group H (Fig. 5h, i). Between groups P and M, 986 KO terms (from 219 pathways) and 32 pathways were significantly changed (*z* > 1.6, enrichment analysis; Additional file 3: Table S3E, H). These pathways were all group-P enriched, except for glycosaminoglycan degradation (depleted; 7/11). In the carbon metabolism, probiotics intake leads to higher abundance of galactose metabolism (28/42), pyruvate metabolism (43/64), and glycolysis/gluconeogenesis (42/59). Pathways for intestinal mucosa repair and inflammatory suppression, including butanoate metabolism (31/45), propanoate metabolism (31/44), and selenocompound metabolism (15/18), were also stimulated by probiotics. Furthermore, bacterial chemotaxis (22/24) and flagellar assembly (35/36) became more abundant. These features between groups P and M are mostly consistent or conserved with the health-enriched features identified from the comparison between groups M and H (Fig. 5j), although a few non-conserved pathways related to vitamin B such as biotin metabolism (12/14), one carbon pool by folate (16/18), and pantothenate and CoA biosynthesis (21/23) were specifically enriched in group P versus group M. Collectively, these results suggest that the probiotic intake led to a functional shift of murine intestinal microbiota toward the healthy state, plus a significant degree of host-symptom relief, despite the lack of conservation in taxonomical structure between groups H and P (Fig. 5k).

OTU-KO correlation analysis revealed that the Bacteroidetes OTUs that distinguish group P from group M (which were less abundant in group P and represented 31% of total differential OTUs) were positively correlated with glycosaminoglycan degradation (Fig. 4e). For example, in the Bacteroidetes cluster, the group P-depleted KOs of K01565, K01132, and K01135 were all implicated in glycosaminoglycan degradation (Fig. 4e). Considering that glycosaminoglycan degrading activities were much higher in group M than in group H, it is possible that the probiotic intake reduced the extent of glycosaminoglycan degradation by inhibiting selected Bacteroidetes OTUs that underlie such degradative activities.

### Amplification of disease effect by microbiota transplantation across two orders of mammals

**Fig. 6:**
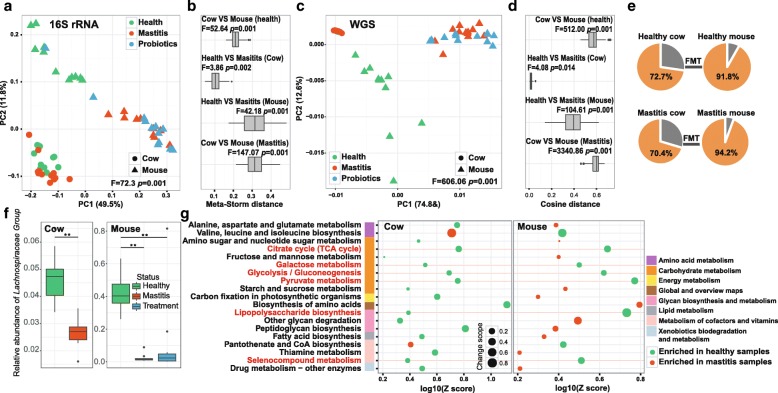
Comparison of mastitis-associated microbiota in cow and those in mouse. PCoA clustering (**a**) and relative similarity (**b**) of bovine and murine microbiota based on organismal structure (via Meta-Storm distance) were shown. Moreover, PCA (**c**) and relative similarity (**d**) based on functional gene structure (via cosine distance of KOs) were presented. **e** Unique and shared OTUs and KOs before and after FMT in cows and mice. **f** OTUs that were shared between cows and mice during the FMT from healthy cows to healthy mice (upper panel) or that from diseased cows to diseased mice (lower panel). Red-font highlighted are the mastitis-associated OTUs in mice. **g** Mastitis-associated pathways that were shared between cows and mice. Those that were enriched in both cows and mice were highlighted in red fonts

The ability to recapitulate mastitis in germ-free mice via FMT from mastitis cows supports gut microbiota as a cause, instead of consequence, of mastitis. Interestingly, the cross-mammal-order microbiota transplantation resulted in not just conservation in mastitis symptom but also amplification of intestinal microbiota dysbiosis, as evidenced in the 3-fold amplification of divergence (OTUs) between healthy and mastitis microbiota (averaged distance of 0.104 between groups H and M in cow versus 0.312 in mouse; Fig. 6a, b) and 23-fold amplification of averaged distance (KOs) between healthy and mastitis (averaged distance of 0.017 between groups H and M in cow versus 0.389 in mouse; Fig. 6c, d).

We next probed how such “amplification effect” occurred and in particular, why the very large difference between donor microbiota and xenomicrobiota ended up with similar disease outcome. In the gut microbiota of the murine recipients, majority of family-level taxa (91.8% for healthy pairs and 94.2% for mastitis pairs) were from those of the cow donors (Fig. 6e). However, only one genus-level taxon, of *Lachnospiraceae Group*, exhibited identical trend of enrichment (i.e., enriched in healthy microbiota as compared to diseased ones) between cow and mouse (Fig. 6f). Although *Lachnospiraceae Group* represented only 3% of bacterial abundance in mastitis cows and mastitis mice, they were predominant in healthy murine gut (40%, as compared to 5% in healthy bovine gut). Thus loss of *Lachnospiraceae* may be associated with mastitis and *Lachnospiraceae* appeared to be critical to a healthy host state.

**Fig. 7:**
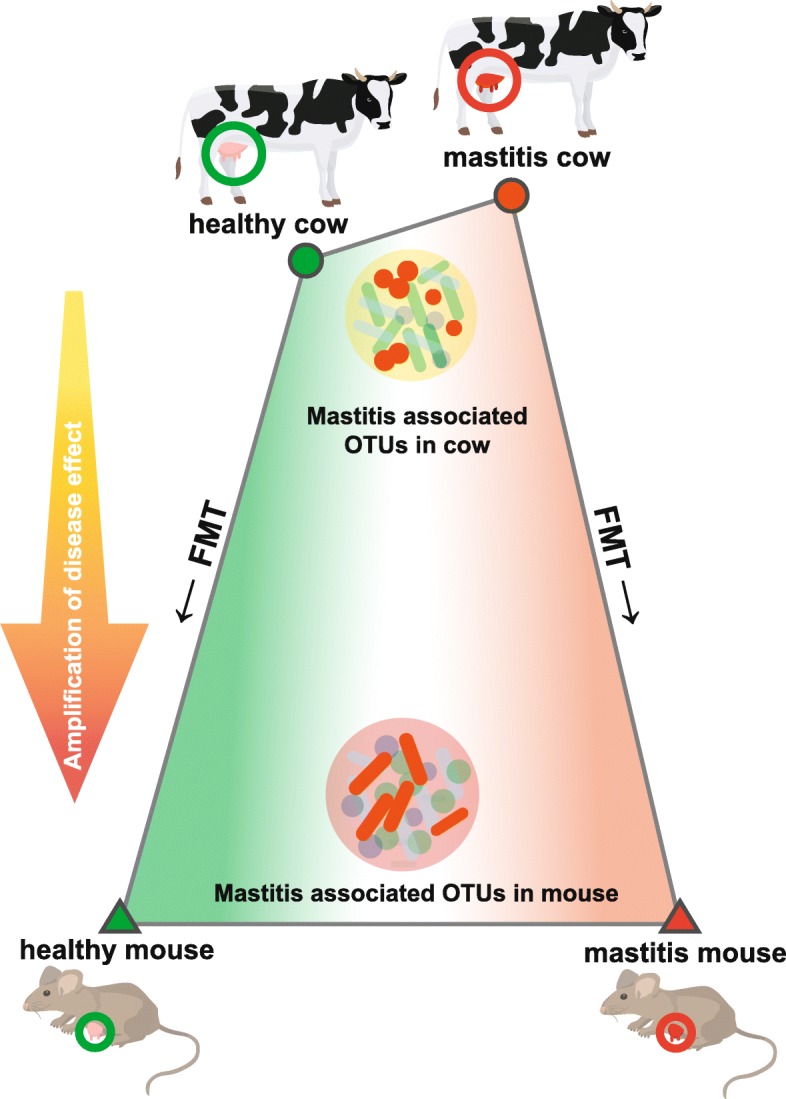
Amplification of disease effect on intestinal microbiota by cow-to-mouse FMT. In the cow-to-mouse FMT, recapitulation of mastitis symptom was accompanied by amplification of the distinction between healthy and diseased microbiota, plus a dramatic change of mastitis-associated OTUs and microbial functions

From the functional perspective, between the 269 mastitis-associated KOs (and 42 such pathways) in cow and the 3525 mastitis-associated KOs (and 25 such pathways) in mouse, 83 KOs (and 6 pathways) are shared that also showed an identical trend of alteration between diseased and healthy hosts. These six pathways are all of lower abundance in mastitis, including TCA cycle, galactose metabolism, glycolysis/gluconeogenesis, pyruvate metabolism, lipopolysaccharide biosynthesis, and selenocompound metabolism. Notably, the degree of enrichment (*Z* score) for these six pathways was each higher in mice than in cows (Fig. 6g). On the other hand, the vast majority (93.5% in cow and 98.4% in mouse) of disease-associated KOs are not shared between cow and mouse. Moreover, distinction in the disease-associated KOs was profound: those in cow featured disease-specific enrichment of certain vitamin B metabolism pathways (Fig. 1f), while those in mouse were characterized by disease-specific depletion of bacterial chemotaxis and flagellar assembly (Fig. 4e). Together, these results suggest that in the cow-to-mouse FMT, recapitulation of mastitis symptom was accompanied by amplification of the distinction between healthy and diseased microbiota, plus a dramatic change of mastitis-associated OTUs and microbial functions (Fig. 7).

## Discussion

In this study, full-length 16S rRNA analysis based on single-molecule sequencing platform was employed for profiling the taxonomic structure of bovine gut microbiota and the subsequently derived mouse gut microbiota, since the number and quality of reference genomes from bovine gut microbiota is much lower than that from either human and mice (in fact, on average ~ 30% of shotgun metagenomic reads in our samples failed to find qualified matches in the RefSeq database of Genbank). In addition, shotgun metagenome sequencing was performed for functional profiling of the microbiota. Although 16S amplicon-based and shotgun metagenome-based taxonomic analyses can sometimes produce distinct landscape of microbial diversity, in this study the results between the two approaches are highly consistent at the species level (Procrustes analysis; Monte Carlo test *p* = 0.0012).

In showing that FMT from diseased, but not healthy cows, caused mastitis in GF mice, our work suggested that bovine mastitis is not necessarily a local infection of mammary glands and in fact can be caused by a dysfunctional intestinal microbiota. Additional support for this finding includes, for example, in mastitis cow a close association between gut microbiota and milk microbiota was observed: the mastitis-associated microbiota change was similar between the two sites, which is characterized by a general increase in *Enterococcus*, *Streptococcus*, and *Staphylococcus* yet deprivation of *Lactobacillus* [30]. On the other hand, known bovine mastitis-associated bacteria such as *S*. *uberis* were not detected in any of the microbiomes of mastitis-active cows or mice, which argue against *S*. *uberis* as the causative agent of mastitis in this study. Notably, each mouse was individually caged while the three groups of mice were physically separated into different gnotobiotic isolators. It is therefore possible for an island effect within each isolator that contributed to the difference in the gut microbiota between the groups of mice [31].

Although our results here did not rule out the role of individual bacteria such as *S*. *uberis* in some mastitis [11], the implication of intestinal microbiota as one potential causes of mastitis might point to a new direction of mastitis treatment and drug development. Intestinal microbiota of mastitis cows are characterized by a wide-ranging and profound change of both organismal and functional profiles, such as the increase of OTUs such as *Oscillospira* spp. and *Ruminococcaceae* spp. and the inhibition of vitamin B metabolism and carbon metabolic activity. However, surprisingly, striking divergence in mastitis-associated intestinal bacteria was evident between cow and mouse, as few mastitis-associated bacterial organismal or functional markers were common between diseased cows and mice, despite conservation in mastitis symptoms. This further supported that, much like FMT of obese human hosts resulted in obesity in germ-free mice [32], mastitis can result from a gut microbiota that is predisposed to mastitis risk. Therefore, restoration of intestinal ecosystem function such as the mastitis-associated pathways identified here can potentially serve as an effective therapeutic strategy for bovine mastitis, which may deserve validation in additional bovine cohorts.

This mouse experiments here support the potential efficacy of probiotic treatment. Mice that consumed probiotics in parallel with FMT from diseased cows exhibited greatly relieved mastitis symptoms plus a molecular immune response that was quite similar to healthy mice, although their intestinal microbiota is structurally similar to those of diseased mice yet functionally distinct from those of not just diseased but healthy mice. It is possible here that probiotics intake treated mastitis via a combination of stimulating host immune system and altering gut microbiota composition. Probiotics can downregulate inflammatory responses in human [33] and animal models [34] and temporally change gut microbiota composition in human. In fact, in human trials, probiotics of lactic acid bacteria can be as efficacious as common antibiotic treatments [35, 36], while avoiding negative consequences of the latter. Specifically, antibiotic therapies that target these presumed pathogens, despite being a common and routine treatment strategy prescribed for lactational mastitis at present [37], can result in residual antibiotics in milk that jeopardizes neonate health [7], such as disrupting normal microbiota development in the digestive and respiratory tracks of breastfed infants. Our work thus provides a theoretical basis for designing and interpreting future trials that target gut microbiota for therapy and even prevention of mastitis, in both dairy animals and human.

Probiotics do not necessarily target restoration of the microbial community, as, for example, some probiotic species increase colonization resistance to pathogens. On the other hand, in this particular case, the intestinal OTUs that were reduced by administration of the probiotic strain *Lactobacillus casei Zhang* are all commensals that were not reported as bovine mastitis-associated pathogens in past studies. Moreover, no *L*. *casei Zhang* were detected on breast surface of recipient mice, which argues against colonization resistance to pathogenic bacteria in breast tissue by the probiotic strain.

Finally, the ability to recapitulate key physiological and immunological features of bovine mastitis in germ-free mice via FMT, plus the fact that mastitis affects nearly all lactating mammals, advocated for mastitis as a new research model to study the co-evolution of gut microbiome, mammalian genomes, and inflammatory diseases. Advantages of the model also include, e.g., relative ease of disease symptom measurement, short time span of disease onset and progression, and ability to intervene disease development. Moreover, mechanistically understanding the “amplification effect” of mastitis-associated microbiota structure and function in cow-to-mouse FMT might shed new light on rational selection of animal models and proper interpretation of the rapidly increasing microbiota transplantation experiments that aim to interrogate microbiota role in chronical diseases.

## Materials and method

### Fecal microbiota sampling and FMT

The study protocol was approved by the Ethical Committee of Third Military Medical University (Chongqing, China). Permission was also obtained from the owners of sampled dairy farm. Every effort was made to minimize animal suffering. Holstein cows of 3 to 6 years old and averaging ~ 600 kg of weight from three dairy farms in Chengdu, Sichuan province were employed as the donors of fecal microbiota. The animals were maintained on standard diet of grass-legume hay that conformed to the daily nutrient requirements for mature lactating cows [38]. For the preceding 2 years, none of the animals had received any treatments involving antibiotics or other drugs. Mastitis was diagnosed based on SCC (the number of leukocytes per milliliter of fresh milk) using a Bentley FTS/FCM400 Combi Instrument (Chaska, USA) [38]. To prevent the milk samples from environmental microbial contamination, the cow udders and teats were wiped with cotton wool soaked in 70% ethanol and the first few streams of milk were discarded before sample collection. The cows with SCC > 2 × 10^6^ cells/mL and with redness and swelling around the udder tissue were diagnosed as mastitis, while those with SCC < 2 × 10^4^ cells/mL and free of clinical mastitis signs such as abnormal milk production, redness, and swelling around the udder tissue were diagnosed as healthy [39]. Fresh fecal samples from 12 mastitis and 12 healthy cows were respectively collected. Aliquots of the samples either proceeded to FMT or were frozen immediately upon collection and then stored at − 80 °C for DNA extraction and sequencing.

For the FMT procedure, all fecal samples were handled under anaerobic conditions. For either the healthy or the mastitis group, fecal samples were freshly collected, the content was thereafter divided into aliquots and frozen in liquid nitrogen and thereafter stored at − 80 °C. At the day of inoculation, 0.5 g fecal sample from each of the cows was mixed together and then suspended with twice the fecal volume of sterile physiological saline. After thorough mixing and resting (to minimize the amount of bacteria lost), the supernatant was collected and FMT was performed by a single oral administration of 1 g/kg fecal suspension [40]. GF mice were provided and housed according to animal care regulations in the germ-free animal facility at Third Military Medical University (Chongqing, China). A total of 35 pregnant female adult (12-week-old; germ-free) C57BL/6J mice were each colonized with 0.3 mL fecal supernatant derived from either healthy cows or mastitis cows. FMT was performed on day 17 after mating, as the murine pregnancy phase lasts 19 to 21 days after mating and the lactating phase immediately followed the delivery. The mice were randomly divided into three groups, which received fecal transplants from (i) healthy cows (group; *n* = 11), (ii) mastitis cows (group M; *n* = 12), or (iii) mastitis cows, plus probiotic administration to these recipient mice (group P; *n* = 12). For group P, the probiotic strain *Lactobacillus casei Zhang* was administered at a dose of 5 × 10^8^ cfu per day for 25 days after FMT (*Lactobacillus casei Zhang* was a probiotic strain isolated from traditional homemade koumiss in Inner Mongolia of China; it was previously shown to exhibit anti-oxidative and anti-inflammatory effects in rats [41, 42]). During the same period, groups H and M were fed normal saline as a vehicle control, at identical volume and time points as the probiotic in group P. In order to prevent cross-contamination of gut microbiota, the three groups of mice were physically separated into different gnotobiotic isolators after FMT; moreover, each mouse was housed in a separate cage with safe distance apart within each of the individual gnotobiotic isolator, so as to prevent any island effects. At the end of day 25, the mice were sacrificed and fresh fecal samples collected, followed by immediate addition of Sample Protector for RNA/DNA (Takara Japan) and then stored at − 80 °C before sequencing.

### Breast surface infection experiment

In addition to the groups above, three mice were separately raised for the surface infection experiment. On the third day after FMT with healthy cow feces, 0.5 g fecal sample from each of the mastitis cows was mixed together and then suspended with twice the fecal volume of sterile physiological saline. After thorough mixing and resting (to minimize the amount of bacteria lost), 2 mL of the supernatant was collected and gently smeared on the breast surface of the three mice by swabs.

### Histopathological analysis

For mice, collection of milk for SCC measurement was not feasible, therefore histopathological examination was employed to assess the alterations and inflammation of mammary gland tissue during mastitis [20]. Mammary gland, small intestine, and colon tissues were fixed in 4% paraformaldehyde for at least 48 h and embedded in paraffin wax. Sections were deparaffinized with xylene and gradually rehydrated through graded alcohols for staining. Sections were stained with hematoxylin and eosin (i.e., HE staining), and then examined under a light microscope [43]. HE-stained sections of mammary gland tissues were reviewed manually first and representative sections for each group were selected and processed further for immunohistochemical analysis.

The primary antibody used for section staining was a rabbit IgG polyclonal antibody specifically for mouse CD45 at 1:200 dilutions. CD45 was chosen because as the first and prototypic receptor-like protein tyrosine phosphatase, it is expressed on all nucleated hematopoietic cells and plays a central role in adaptive immunity [22]. The staining procedure was mostly as past described [44, 45]. Due to an apparent increase in sensitivity of immune cells that infiltrated to mammary gland tissue to experimental processing, extra care was taken to protect the integrity of the immune cells and avoid their disruption. The degree of jejunum and colon injury was assessed via the Chiu Scoring System [24] in a blinded manner, where the number of inflammatory cells was counted in 12 randomly selected fields from each slide at a magnification of × 400. The degree of necrosis in mammary gland tissues was scored on a scale of 0 to 3 (normal 0, mild 1, moderate 2, severe 3). The degree of jejunum and colon injury was scored as grades 0 (normal mucosa), 1 (development of subepithelial spaces at villus tips), 2 (extension of the subepithelial space with moderate lifting of the epithelial layer), 3 (massive epithelial lifting with a few denuded villi), 4 (denuded villi with exposed capillaries), and 5 (disintegration of the lamina propria, ulceration, and hemorrhage). The liver injury score was recorded via a scale of 0 to 3 (normal 0, mild 1, moderate 2, severe 3), where individual liver sections were evaluated for steatosis, hepatic cellular infiltration, oncotic necrosis, apoptosis, lobular inflammation, and ballooning degeneration using previously defined criteria [46, 47].

### Inflammatory cytokines assay

Seven mice from each of the H, M, and P groups were randomly selected for quantification of inflammatory cytokines detection. Every tissue sample from each of the animals was analyzed separately. Specifically, 0.1 g mammary gland tissue was homogenized with 1 mL physiological saline, while 0.04 g each of jejunum, colon, and spleen tissues was homogenized with 400 μL physiological saline. Serum was diluted for five times before assays. All the procedures were performed on ice. After centrifugation at 12,000 rpm for 15 min at 4 °C, supernatant was collected and assayed for IFN-y, TNF-α, IL-1β, IL-4, IL-6, IL-10, IL-17, lysozyme, endotoxin, and myeloperoxidase (MPO) secretion levels using enzyme-linked immunosorbent assay (ELISA) kits. All the antibodies and ELISA kits were from Abcam (UK) and Biolegend (USA) unless specified otherwise.

### Western blot analysis

Mammary gland tissues were homogenized on ice for analysis of NF-κb, STAT3, CLC4, ERK, Akt, Iκb-β, p38, TGR, and JNK protein levels. Total proteins were extracted from 100 mg mammary gland tissues from each group, and concentration was determined by BCA protein assay. Equal amounts of 100 μg total proteins were loaded into each well and fractionated on a 10% SDS polyacrylamide gel. The housekeeping gene of β-actin was used as an internal control for assessing equal loading of total protein among wells. Abundance of target protein was quantified using an enhanced chemiluminescence detection system.

### Full-length 16S rRNA gene sequencing using PacBio

The full-length 16S rRNA gene extracted from the fecal samples of cows and recipient mice at 25 days post-FMT were sequenced using PacBio RS II (Pacific Bioscience, USA), as the long-read sequencing was shown to improve OTU quality and decrease variance [48]. Specifically, fecal samples were pulverized with a mortar and pestle in liquid nitrogen, and bacterial genomic DNA was extracted by the Power Soil DNA Isolation Kit (MoBio, USA). The bacterial 16S rRNA was amplified by PCR for barcoded SMRT sequencing with the forward primer 27F (5′-GAGAGTTTGATCCTGGCTCAG-3′) and the reverse primer 1541R (5′-AAGGAGGTGATCCAGCCGCA-3′). These primers contained a set of 16-nucleotide barcodes. The library size was confirmed on a Tape station (Agilent, USA) before PacBio SMRT sequencing. Raw sequences were initially processed via the PacBio SMRT portal (version 2.7, Pacific Bioscience, USA). Sequences were filtered for a minimum of one, two, four, and eight passes, and a minimum predicted accuracy of 90%. Sequences of < 1400 bp and > 1800 bp as well as those containing any primer mismatches, barcode mismatches, ambiguous bases, and homopolymer runs exceeding six bases were excluded. A total of 664,284 high-quality 16S rRNA gene sequences were obtained, with 11654 ± 4663 reads per fecal sample (Additional file 1: Table S1). Downstream bioinformatics analysis was performed using Parallel-Meta 3, a software package for comprehensive taxonomical and functional comparison of microbial communities [49], from which operational taxonomic unit (OTU) tables were derived. Alpha diversity was calculated by four different parameters: (i) observed OTUs, (ii) Shannon Index, (iii) Simpson Index, and (iv) Chao1 index. Distance matrices between samples were computed based on weighted Meta-Storm algorithms [15].

### Metagenomic sequencing and functional gene-based analysis

Paired-end metagenomic sequencing was performed for the intestinal microbiota from each of the 12 healthy cows, 12 mastitis cows, 12 healthy mice, 11 mastitis mice, and 12 probiotics intake mice, via the Illumina HiSeq 2500 platform, yielding 25.2 ± 2.34 Gb per sample (paired-end reads, with average fragment insert size of 350 bp and average read length of 150 bp). The reads were quality controlled by Trimmomatic [50] (Sliding window 4:20; Minlength 100; MinPhred 25; Percentage of MinPhred 80, remaining 22,901,946 reads per sample, SD 6,570,335) and de novo assembled into contigs via SPAdes v3.7.1 with default parameters (except “-meta”) [51].

Gene prediction from the assembled contigs was performed using GeneMark v2.7d [52]. Relative abundance of the genes was determined by aligning high-quality sequencing reads to the gene catalog using Bowtie2 [53] and Samtool [54]. Putative amino acid sequences identified from the contigs were then aligned against the proteins/domains in the KEGG databases via KAAS (http://www.genome.jp/tools/kaas/).

For KEGG enrichment analysis, the *Z* score was used as the final reporter score for evaluating the enrichment of specific pathways [55]. A *Z* score of 1.6 (90% confidence according to normal distribution) was used as a detection threshold for significantly differentiating pathways. Whether a pathway was upregulated or downregulated was determined by Wilcoxon rank-sum test (*p* < 0.05). If both upregulated and downregulated KOs were present in a pathway, the pathway was considered upregulated only when the number of upregulated KOs was at least 10% more than that of downregulated KOs, and vise versa. All the *p* values in this paper were BH corrected and *q* < 0.1 was used as threshold.

### Analysis of OTU-KO networks

In both cow and mouse, potential links between mastitis-associated genes and OTUs were identified using co-occurrence analysis. Specifically, in comparison of the fecal microbiota between healthy controls and mastitis hosts, one-tail Wilcoxon rank-sum test was performed on all the OTUs or KOs that occurred in more than five samples and adjusted for multiple testing using the Benjamin-Hochberg procedure (*q* < 0.01). The differential OTUs and KOs identified were then further clustered via Spearman’s correlation (*r* > 0.8) between their abundances in all samples by R (3.2 ccrepe package). The co-occurrence network was visualized and adjusted by Cytoscape.

### Probiotic administration

The administered probiotics of *Lactobacillus casei Zhang* was isolated from traditional homemade koumiss in Inner Mongolia of China [56], and its complete genome has been published by the authors’ lab [41]. In our study, the strain-specific primers for *L*. *casei Zhang* (LcZ-F: CCGACGTACCAGCTCACT; LcZ-R: AAGACTATCAGATAGCGGCTCA [57]) were employed to investigate the colonization in the mice gut and breast surface. PCR results showed that gastric administration did allow *L*. *casei Zhang* to colonize the intestine of mice in each of the corresponding samples (genomic sequences of this strain was also detected in the shotgun metagenome data), while on the breast surface the strain was not detected.

## Additional files

Additional file 1: **Table S1.** General information of the FMT donors (cow) and recipients (mouse). (XLSX 12 kb)

Additional file 2: **Table S2.** A. Differential OTUs between mastitis and healthy cows. B. Differential OTUs between Group H and Group M in mice. C. Differential OTUs between Group M and Group P in mice. (XLSX 19 kb)

Additional file 3: **Table S3.** A. Relative abundance of identified KOs in cows. B. Relative abundance of identified KOs in mice. C. Differential KOs between mastitis and healthy cows. D. Differential KOs between Group H and Group M in mice. E. Differential KOs between Group M and Group P in mice. F. Enriched pathways between mastitis and healthy cows. G. Enriched pathways between Group H and Group M in mice. H. Enriched pathways between Group M and Group P in mice. (XLSX 2986 kb)

Additional file 4: **Table S4.** A. Spearman coefficients between differential OTUs and KOs in cows. B. Spearman coefficients between differential OTUs and KOs in mice. (XLSX 51 kb)

Additional file 5: **Figure S1.** Representative photomicrographs of hematoxylin-eosin stained mammary gland tissue (200 X magnification) of (a) healthy mice and (b) mastitis-active mice. (c~e) Pathological changes in mammary gland surface of the three mice which were transplanted with healthy cow feces and administered with mastitis cow feces on their breast surface. No inflammation was present in the mammary glands of these three mice throughput the duration of experiment. (PDF 2369 kb)

Additional file 6: **Table S5.** Level of inflammatory cytokines in various mouse tissues as measured by ELISA. (XLSX 10 kb)
