# Supplementary figures and images for: Cow-to-mouse fecal transplantations suggest intestinal microbiome as one cause of mastitis
Source: Microbiome. 2018 Nov 8;6:200. doi: 10.1186/s40168-018-0578-1 (PMC6225715; doi:10.1186/s40168-018-0578-1)

**Figure S1**

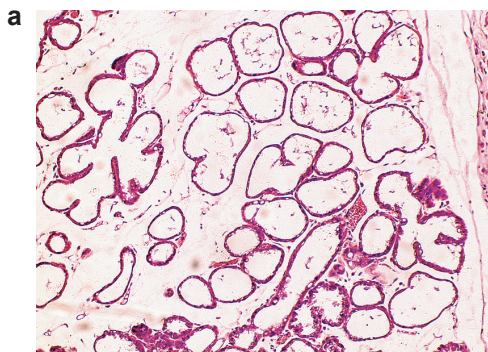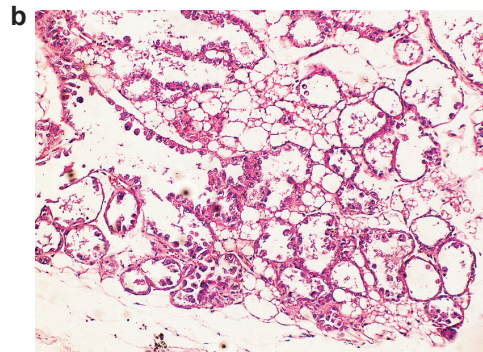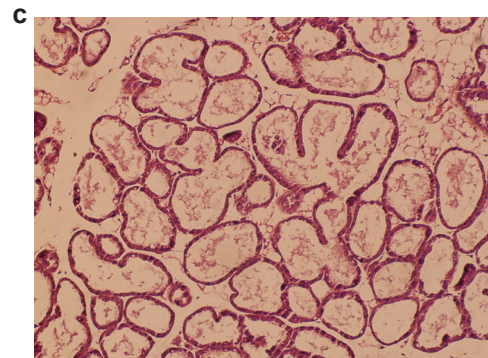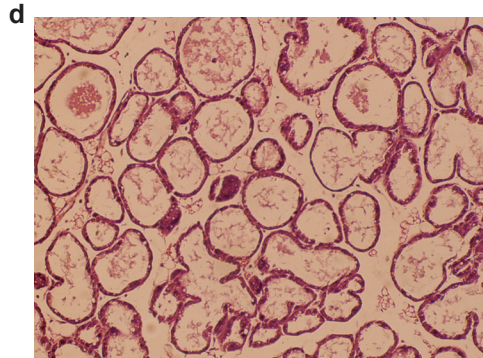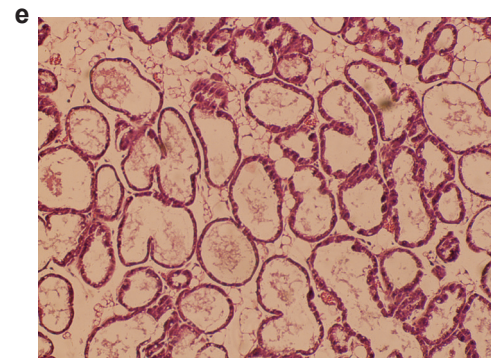

Supplement: Supplementary file 5 — Figure S1. Representative photomicrographs of hematoxylin-eosin stained mammary gland tissue (200 X magnification) of (a) healthy mice and (b) mastitis-active mice. (c~e) Pathological changes in mammary gland surface of the three mice which were transplanted with healthy cow feces and administered with mastitis cow feces on their breast surface. No inflammation was present in the mammary glands of these three mice throughput the duration of experiment. (PDF 2369 kb) [file 40168_2018_578_MOESM5_ESM.pdf]
